# Studies on CYP3A activity during the menstrual cycle as measured by urinary 6β‐hydroxycortisol/cortisol

**DOI:** 10.1002/prp2.884

**Published:** 2021-10-19

**Authors:** Helena Bergström, Anna Lindahl, Anna Warnqvist, Ulf Diczfalusy, Lena Ekström, Linda Björkhem‐Bergman

**Affiliations:** ^1^ Department of Neurobiology Care Sciences and Society (NVS) Division of Clinical Geriatrics Karolinska Institutet Huddinge Sweden; ^2^ Department of Laboratory Medicine Division of Clinical Chemistry Karolinska Institutet Stockholm Sweden; ^3^ Department of Clinical Chemistry Karolinska University Laboratory Karolinska University Hospital Stockholm Sweden; ^4^ Department of Environmental Medicine Division of Biostatistics Karolinska Institutet Stockholm Sweden; ^5^ Department of Laboratory Medicine Division of Clinical Pharmacology Karolinska Institutet Karolinska University Hospital Stockholm Sweden; ^6^ Department of Palliative Medicine Stockholms Sjukhem Stockholm Sweden

**Keywords:** 6β‐hydroxycortisol/cortisol, CYP3A activity, menstrual cycle, miRNAs, TMAO

## Abstract

The 6β‐OH‐cortisol/cortisol ratio (6β‐OHC/C) in urine is an endogenous marker of drug‐metabolizing enzyme cytochrome P450 3A (CYP3A). The primary aim of this single center, prospective, non‐interventional cohort study, was to investigate the variability of 6β‐OHC/C during the menstrual cycle. In addition, possible associations between the CYP3A activity and sex hormones, gut microbiota metabolite trimethylamine‐N‐Oxide (TMAO) and microRNA‐27b, respectively, were investigated. Serum and urinary samples from healthy, regularly menstruating women followed for two menstrual cycles were analyzed. Twenty‐six complete menstrual cycles including follicular, ovulatory, and luteal phase were defined based on hormone analyses in serum. 6β‐OHC/C were analyzed in urine and sex hormones, TMAO and miRNA‐27b were analyzed in serum at the same time points. 6β‐OHC/C did not vary between the follicular, ovulatory, or luteal phases. There was a difference in the relative miRNA‐27b expression between the follicular and ovulatory phase (*p* = .03). A significant association was found between 6β‐OHC/C and progesterone during the follicular (*p* = .005) and ovulatory (*p* = .01) phases (*n* = 26 for each phase). In addition, a significant association was found between the ratio and TMAO during the ovulatory (*p* = .02) and luteal (*p* = .002) phases. 6β‐OHC/C and gut microbiota TMAO were significantly associated (*p* = .003) when evaluating all values, for all phases (*n* = 78). Interestingly, the finding of an association between 6β‐OHC/C in urine and levels of TMAO in serum suggest that gut microbiota may affect CYP3A activity.

## Graphical abstract

A significant association between 6β‐OHC/C and the microbiota metabolite TMAO was noted in this study, suggesting that gut microbiota may affect CYP3A activity.

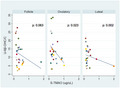

**Abbreviations**
6β‐OHC/C: 6β‐hydroxycortisol to cortisol
ABP: Athlete Biological Passport
CYP3A: cytochrome P450 3A4
FSH: follicular stimulating hormone
LH: luteinizing hormone
miRNA: microRNA
TMAO: trimethylamine‐N‐Oxide

## 1 INTRODUCTION

Cytochrome P450 3A (CYP3A) is the major drug‐metabolizing enzyme in the liver and the gut, metabolizing more than 50% of all drugs. CYP3A also participate in the metabolism of endogenous steroids and xenobiotics. Factors like age, ethnicity, diet, inflammatory disease, and alcohol intake influence the CYP3A activity.^1^, ^2^, ^3^ In addition, the expression of CYP3A is tissue‐ and sex specific, and it has been shown that women have higher levels of CYP3A mRNA and protein in the liver compared to men.^4^, ^5^

Phenotyping of CYP3A activity using midazolam clearance in plasma is the golden standard for studies using an exogenous marker.^6^ However, there is a currently a lack of consensus concerning sex differences in CYP3A activity using exogenous probes.^7^

The menstrual cycle variability of midazolam has been explored in three studies. However, while one study of 1‐hydroxylation of midazolam showed an increase during the follicular phase, two other studies of midazolam clearance did not demonstrate any differences between phases.^8^, ^9^, ^10^ Regarding investigations of potential menstrual cycle variability in endogenous markers of CYP3A, studies are sparse and the results are difficult to interpret due to methodological issues.^9^, ^10^, ^11^, ^12^, ^13^, ^14^ One of the endogenous markers used to detect CYP3A activity is the 6β‐OH‐cortisol/cortisol (6β‐OHC/C) ratio in urine since cortisol is a substrate for CYP3A.^15^ The advantage with 6β‐OHC/C is its short half‐life that can detect rapid changes in CYP3A activity.^16^ In contrast, another endogenous marker, 4β‐OH‐cholesterol/cholesterol ratio (4β‐OHC/C) measured in plasma, has a long half‐life of more than 2 weeks and reflects CYP3A activity over longer time period.^17^, ^18^ Thus, this marker is less suitable for detecting short‐term changes in CYP3A activity.

In one study exploring menstrual cycle variability of 6β‐OHC/C, cycle phase was determined by a single sample of progesterone in blood^19^ and in other studies on patient narrative or first day of bleeding.^20^, ^21^, ^22^, ^23^ At the same time, in a study of plasma 6β‐OHC/C in 31 women, menstrual cycle phase was not mentioned.^24^ Finally, few studies have evaluated urine 6β‐OHC/C variability in the basal state, as most studies were performed during induction or inhibition of CYP3A.^17^, ^25^ Thus, the question of variability of 6β‐OHC/C during the menstrual cycle phases remains unanswered.

Intestinal microbes, the microbiota, functions like an endocrine organ producing bioactive amines, that may affect host physiology.^26^ The most well‐known representative, is the metabolite trimethylamine‐N‐Oxide (TMAO), that has been shown to be strongly associated with cardiovascular disease.^27^ In addition, it has been suggested that microbiota influence the CYP3A activity in the gut^28^ and the liver.^29^ Microbiota may affect the homeostasis between primary and secondary bile acids, and in this manner, nuclear receptors regulating CYP3A expression are affected.^28^, ^29^ To our knowledge, the association between TMAO and CYP3A activity assessed as 6β‐OHC/C has not been studied before.

MicroRNAs are small, noncoding RNAs that, among other things, have been shown to modulate the expression of drug‐metabolizing enzymes.^1^ Micro RNA‐27b (miRNA‐27b) has been suggested to negatively regulate CYP3A mRNA‐ and protein expression in vitro.^30^ This finding has been confirmed in a small clinical study where miRNA‐27b levels in serum correlated with CYP3A activity in serum.^31^ Furthermore, this association was also found in the liver, however, not in the same individuals. To our knowledge, possible variation of miRNA‐27b has not been studied during menstrual cycle before.

6β‐OHC/C in urine is not yet approved for formal drug–drug interaction (DDI) studies.^32^ However, with the aim of increasing the number of DDI studies performed in vulnerable populations like fertile women of childbearing age, there is a need for more information on this endogenous marker.

Thus, the primary objective of this pilot study was to test the hypothesis that there is a variability of CYP3A activity during the menstrual cycle that could be detected by 6β‐OHC/C using repeated single‐spot urine samples during two menstrual cycles in healthy volunteers. The secondary objectives were to study possible factors that may affect the CYP3A activity during the menstrual cycle including levels of sex hormones, the microbe metabolite TMAO and miRNA‐27b.

## 2 MATERIALS AND METHODS

### 2.1 Description of study population and study design

The power calculation of 30 subjects in this study, was based on the number of subjects needed to show an association between menstrual cycle phases and urinary epitestosterone used in the Athlete Biological Passport (ABP). As data on intraindividual variability of 6β‐OHC/C for CYP3A in women have been inconsistent, an accurate power calculation based on this endpoint was not feasible. Most previous studies on menstrual cycle variability using midazolam as a probe, have included seven to at the most 16 subjects, and there are no studies on 6β‐OHC/C and menstrual cycle variability, thus 30 seemed an appropriate number.^9^, ^10^, ^12^, ^13^ This evaluation was strengthened by the longitudinal study design, with repeated measurements for 6–8 weeks in the same subjects.

Unfortunately, we were not able to recruit more than 19 subjects, and as two subjects (11 and 19) withdrew due to lack of time to participate, the final number of subjects was 17. The study was conducted at the outpatient clinic at the Department of Clinical Pharmacology, Karolinska Hospital, Stockholm, Sweden. Clinical trial registration was not performed as this was a small, observational study.

Thus, in this single center, prospective, non‐interventional pilot study, 17 healthy regularly menstruating women aged 18–45 years, were followed during two consecutive menstrual cycles. The mean age was 33.2 years, BMI 21.6, bleeding 4.9 days, and cycle length 26.9 days. In addition, the mean number of blood samples were 7.3. Further information on study cohort demographics and a figure of study design, can be found in the articles describing the study results for the ABP by Mullen et al. and Schulze et al.^33^, ^34^ Results on paired lipid and TMAO analyses can be found in an article by Bergström et al.^35^

Morning urine spot samples were collected three times weekly and blood samples were collected once a week. Urine samples to be analyzed for steroids included in the ABP, were treated separately. The aim was that urine samples to be analyzed for 6β‐OHC/C in our part of the study, were samples collected on the same day as blood sampling of hormones once a week. Menstrual cycle phase for each blood sample was determined using an algorithm.^36^ A selection of six urinary samples for each subject, two for each menstrual cycle phase (follicular, ovulatory, and luteal), were to be analyzed for 6β‐OH‐cortisol and cortisol. Additionally, for two subjects (subject 5 and 19) with long menstrual cycles based on narrative, all urine samples collected three times a week, were to be analyzed for 6β‐OHC/C to evaluate the intraindividual variability with a higher number of samples. Blood samples were taken once a week also for these subjects.

Serum samples for miRNA extraction were selected to represent one menstrual cycle including the three phases per subject. Finally, urine and serum samples were either analyzed the same day as sampling, or frozen and stored at −70 until analysis.

### 2.2 Analysis of sex hormones, safety parameters, and TMAO

Estrogen, progesterone, follicle stimulating hormone (FSH), luteinizing hormone (LH), and routine safety parameters were analyzed in the subjects. Methods for the analyses are described in the cited articles.^33^, ^34^, ^35^ Analysis of TMAO in human plasma using LC–MS/MS, was performed at the Swedish Metabolomic Centre in Umeå. Validation of the method is described in the article by Missiliadis et al.^37^

### 2.3 Analysis of cortisol and 6β‐hydroxycortisol using LC–MS/MS

6β‐hydroxycortisol, cortisol, and [^2^H_4_]cortisol were acquired from Sigma‐Aldrich Sweden AB, Stockholm, Sweden. [^2^H_4_]6β‐hydroxycortisol was purchased from Santa Cruz Biotechnology, Dallas, United States.

6β‐hydroxycortisol and cortisol in urine were quantified according to a previously described and validated method.^38^ In brief, sample preparation was performed using a Hamilton Microlab STARlet automated liquid handler (Hamilton Company). Urine, quality control samples, and calibrators were diluted 1:1 v/v with water containing internal standards ([^2^H_4]_6β‐hydroxycortisol, 1 µmol/L, [^2^H_4_]cortisol, 0.4 µmol/L). Samples (200 µl) were then applied to an Oasis HLB µElution 96‐well solid phase extraction plate (Waters). Following a wash with 200 µl 5% methanol in water, analytes were eluted with 100 µl acetonitrile/isopropanol 2:3 v/v followed by dilution with water (400 µl). Levels of 6β‐hydroxycortisol and cortisol were determined using a Xevo TQ MS with an electrospray ionization source, interfaced with an Acquity Classic UPLC system (Waters). Samples (5 µl) were separated on an Acquity UPLC BEH Shield RP18 (1.7 µm, 2.1 × 150 mm) column (Waters) held at 55°C, using a linear gradient with 0.2% formic acid (mobile phase A) and acetonitrile (mobile phase B). The gradient starting conditions were 5% B at a flow rate of 0.4 ml/min, which were ramped up to 50% B over a period of 6 min and then increased to 95% B over 0.5 min. 95% B was then maintained for 1 min before re‐equilibration at 5% B for 3 min. The total run time was 12 min per sample. MS/MS data were acquired in negative mode for the detection of 6β‐hydroxycortisol, and positive mode for the detection of cortisol, in the same run. Two MRM transitions, one quantifier‐, and one qualifier transition, were set up for each analyte: 347 → 313 and 347 → 125 for 6β‐hydroxycortisol and 363 → 121 and 363 → 97 for cortisol. Peak integration, calibration, and quantification was performed using the software TargetLynx (Waters).

### 2.4 Analysis of miRNA‐27b

Micro RNA was extracted from serum samples using miRNAs Serum/Plasma Advanced Kit (Qiagen). The microRNA samples were then subject to cDNA conversion using Thermo Fisher cDNA TaqMan™ Advanced miRNA cDNA Synthesis Kit and the cDNA were diluted in accordance with the protocol. The diluted cDNA was used as template in premade TaqMan assays targeting miR27b (assay IDs 478270_mir, Life Technology) using TaqMan™ Fast Advanced Master Mix. The real‐time PCR were conducted on StepOne. To control for unwanted sources of variation, the relative miRNA expression was calculated with the ddCT formula^39^ using miRNA‐26b expression (assay ID 478418‐mir) as a control as recommended by the provider. This method has previously been described and evaluated by the authors.^31^

### 2.5 Statistical analysis

D'Agostino and Pearson normality test was conducted to test for Gaussian distribution of data and arithmetic means. Median, maximum, minimum, and interquartile range (IQR) were calculated for all variables. None of the variables had Gaussian distribution.

As some subjects had 6β‐OHC/C values from two menstrual cycles and some only one, it was necessary to use a statistical analysis considering the intraindividual correlations caused by repeated measurements. Linear regression using clustered robust covariance estimator was therefore used in all linear regression analyses. To evaluate if 6β‐OHC/C differed between the three menstrual phases using linear regression, data were subset to include two phases at the time. Possible associations between urine 6β‐OHC/C and estrogen, progesterone, follicle stimulating hormone (FSH), luteinizing hormone (LH), and TMAO were also assessed using linear regression.

The interindividual differences in 6β‐OHC/C were evaluated using Kruskal–Wallis test. For the intraindividual variability, coefficient of variation (CV), IQR, and median in the ratio in each subject were calculated. For all subjects, mean ratio and CV were calculated.

As the individuals contributed with only one value for each phase for miRNA‐27b, the variability of miRNA between the menstrual phases was evaluated with Wilcoxon sign‐rank test. The association between 6β‐OHC/C and miRNA (*n* = 27) was assessed with Spearman's rank‐order test, for all values only and not for each phase, due to the small sample size.

Analyzes were performed in Stata 15 (2017 Stata Statistical Software: StataCorp LP.). A value of *p* < .05 was considered statistically significant.

## 3 RESULTS

**FIGURE 1:**
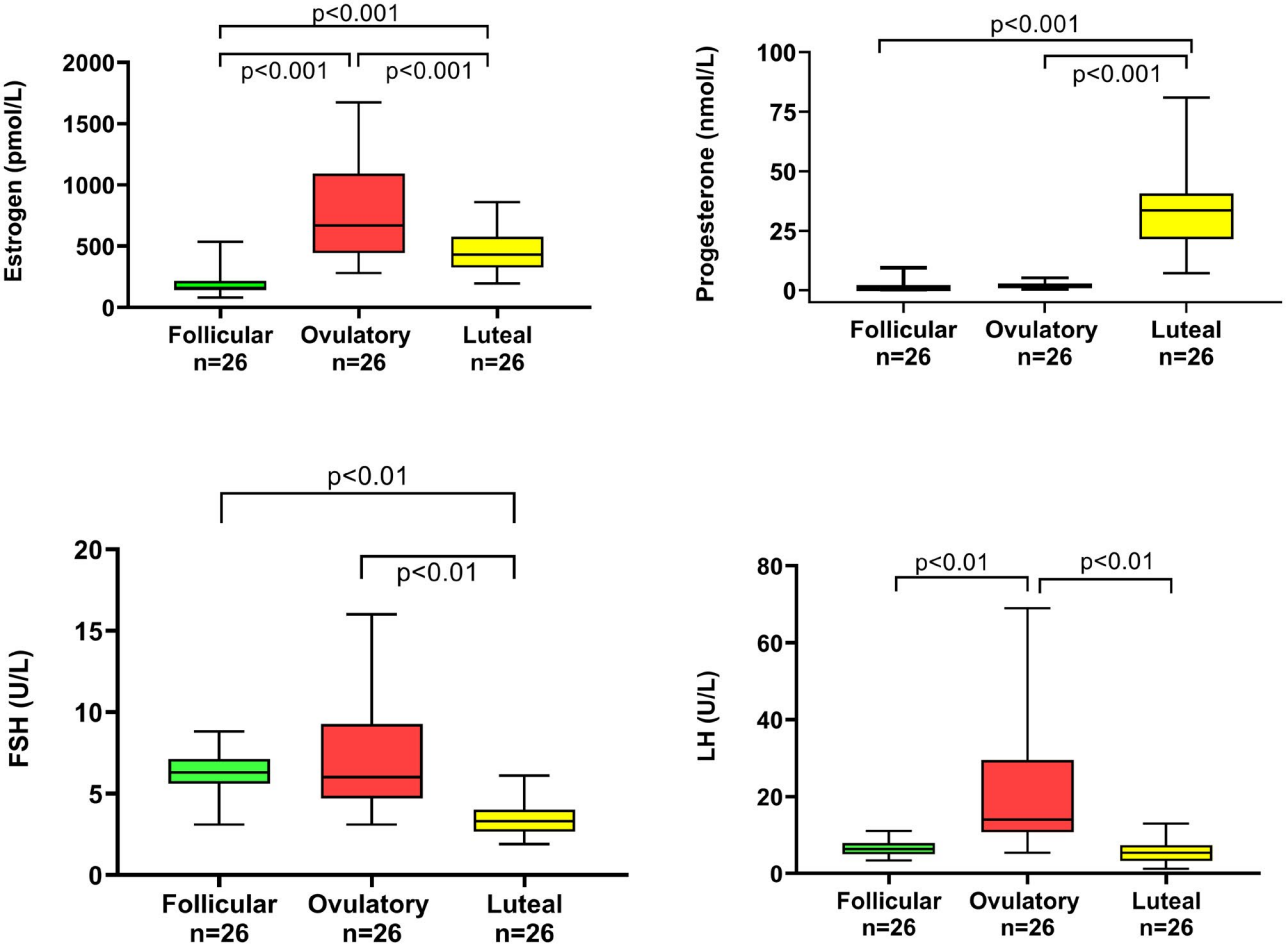
Variations in estrogen, progesterone, follicle stimulating hormone (FSH), and luteinizing hormone (LH) during 26 menstrual cycles from 17 healthy volunteers (*n* = 78)

To confirm known variations in sex hormones and gonadotropins during the menstrual cycle, levels of estrogen and progesterone as well as FSH and LH were controlled (Figure 1). For estrogen, there were significant differences between all phases, whereas progesterone was significantly higher in the luteal phase while FSH was low. As expected, there was a peak in LH during the ovulatory phase.^35^

### 3.1 6**β**‐OH‐cortisol/cortisol samples

Thirteen subjects (subject 5 and 19 included) contributed with six urinary samples of 6β‐OHC/C values and four subjects had three, that is one cycle. Thus, the total number of urinary samples of 6β‐OHC/C was *n* = 90 for evaluating intra‐ and interindividual variability. For the evaluation of intraindividual variability with additional samples, subject 5 contributed with 23 urine samples and 19 with 20 samples.

A total of 78 urine samples could be matched with blood samples analyzed for sex hormones and TMAO. Eleven subjects had six 6β‐OHC/C urinary samples, with matching blood samples (*n* = 66), while three subjects had three urine samples with matching blood samples (*n* = 12). The reason for this discordance, was failure of blood sampling capturing all three phases or that there were urine samples from one menstrual cycle only, that were analyzed. In addition, analyzed urine samples that were taken >2 days before or after the blood sampling, were excluded. The selection of what urine samples to analyze were based on what menstrual phase the subject most likely would be in, based on patient narrative on menstrual cycle and visits.

Thus, altogether 78 urine and blood samples from 26 menstrual cycles (78/3 = 26), were used to evaluate differences in 6β‐OHC/C over the menstrual phases and the associations between 6β‐OHC/C and sex hormones and TMAO, respectively.

### 3.2 6**β**‐OH‐cortisol/cortisol variability

**FIGURE 2:**
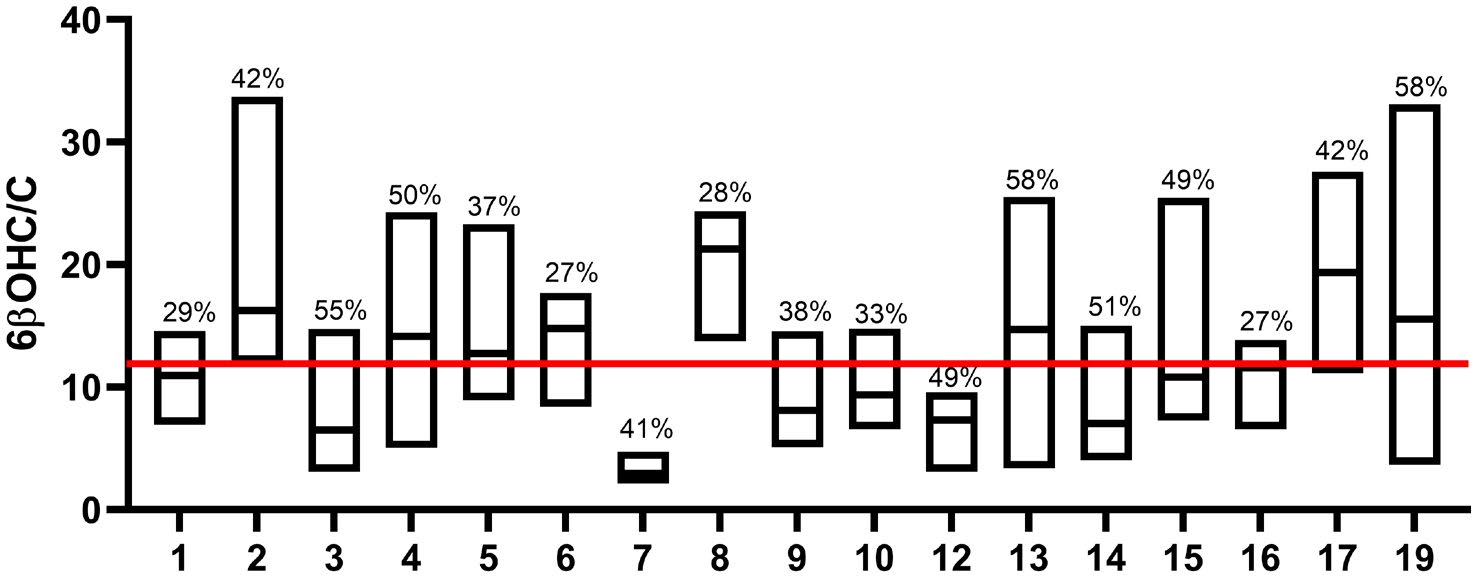
The inter‐ and intraindividual variations in urine 6β‐OH‐cortisol/cortisol (6β‐OHC/C) during two menstrual cycles in 17 healthy volunteers (*n* = 90). The interindividual difference in 6β‐OHC/C was statistically significant (*p* = .002)*. Subject 1,2,3,4,5,6,9,10,13,14,15,16, and 19 (*n* = 13) had six analyzed urine samples, while 7,8,12, and 17 (*n* = 4) had three analyzed urine samples. The figure shows the median, interquartile range (IQR), and coefficient of variation (CV) of 6β‐OHC/C for each subject. The red line shows the mean (12) in 6β‐OHC/C for all subjects (*n* = 90). *Tested by Kruskal–Wallis test

There were significant interindividual differences in the ratio (*n* = 90, *p* = .002**), as evaluated by Kruskal–Wallis test. The intraindividual variability of 6β‐OHC/C was substantial (Figure 2), with mean coefficient of variation (CV) 42%, mean ratio 12 and interquartile range (IQR) 7.43 to 15.17 for all subjects (*n* = 90) (Table S1). For subject 5 and 19, the median in 6β‐OHC/C was 16.93 and 14.63, respectively (Figure S2). CV in the ratio was 33% for subject 5, and 51% for subject 19.

**FIGURE 3:**
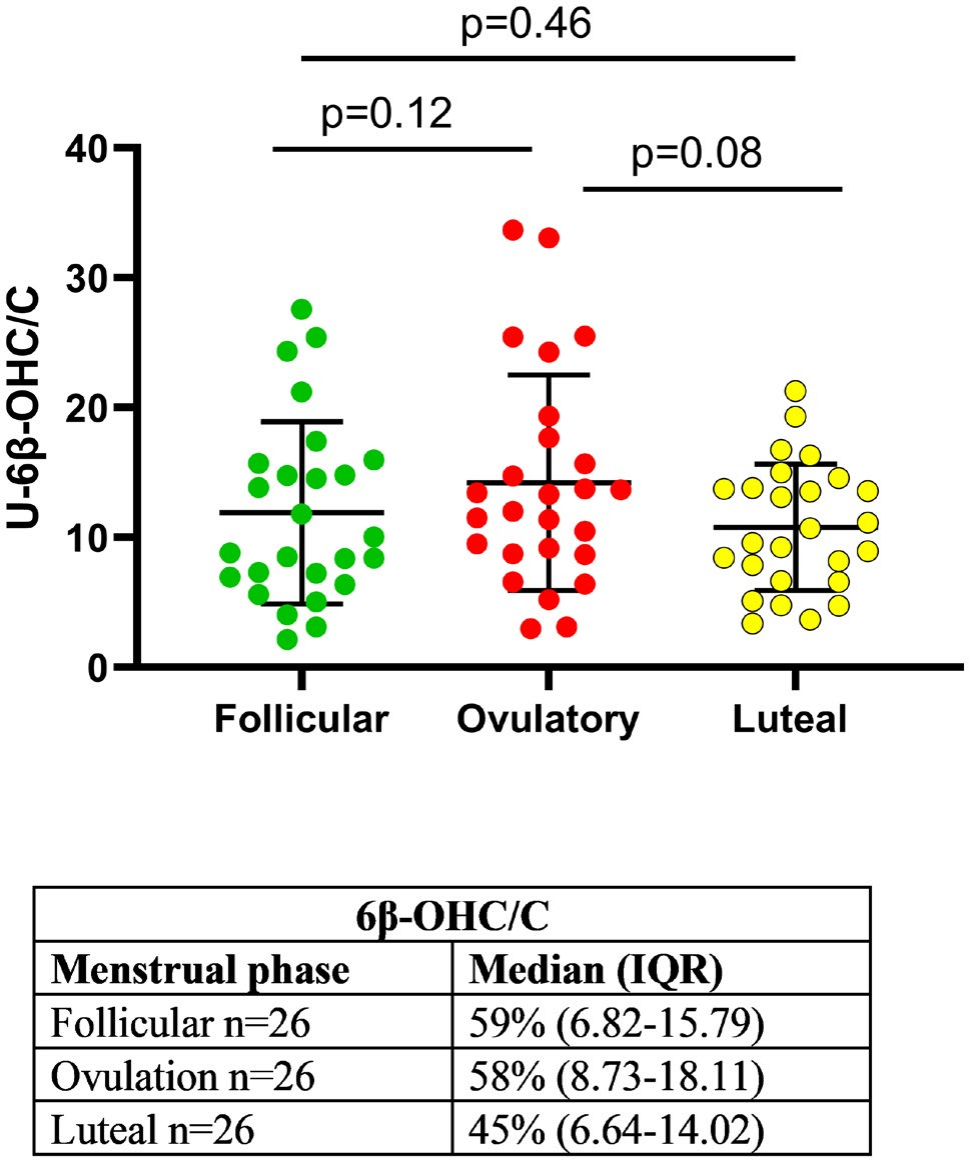
Variations in 6β‐OH‐cortisol/cortisol (6β‐OHC/C) during the three phases of the menstrual cycle including 26 menstrual cycles from 17 healthy volunteers (*n* = 78). The coefficient of variations (CV) in 6β‐OHC/C during the menstrual cycle phases are shown in the table

There were no significant differences in 6β‐OHC/C (*n* = 78) between the follicular, ovulatory, and luteal phases (Figure 3) and no significant difference in the CV between the menstrual cycle phases.

### 3.3 6β‐OH‐cortisol/cortisol and associations

**FIGURE 4:**
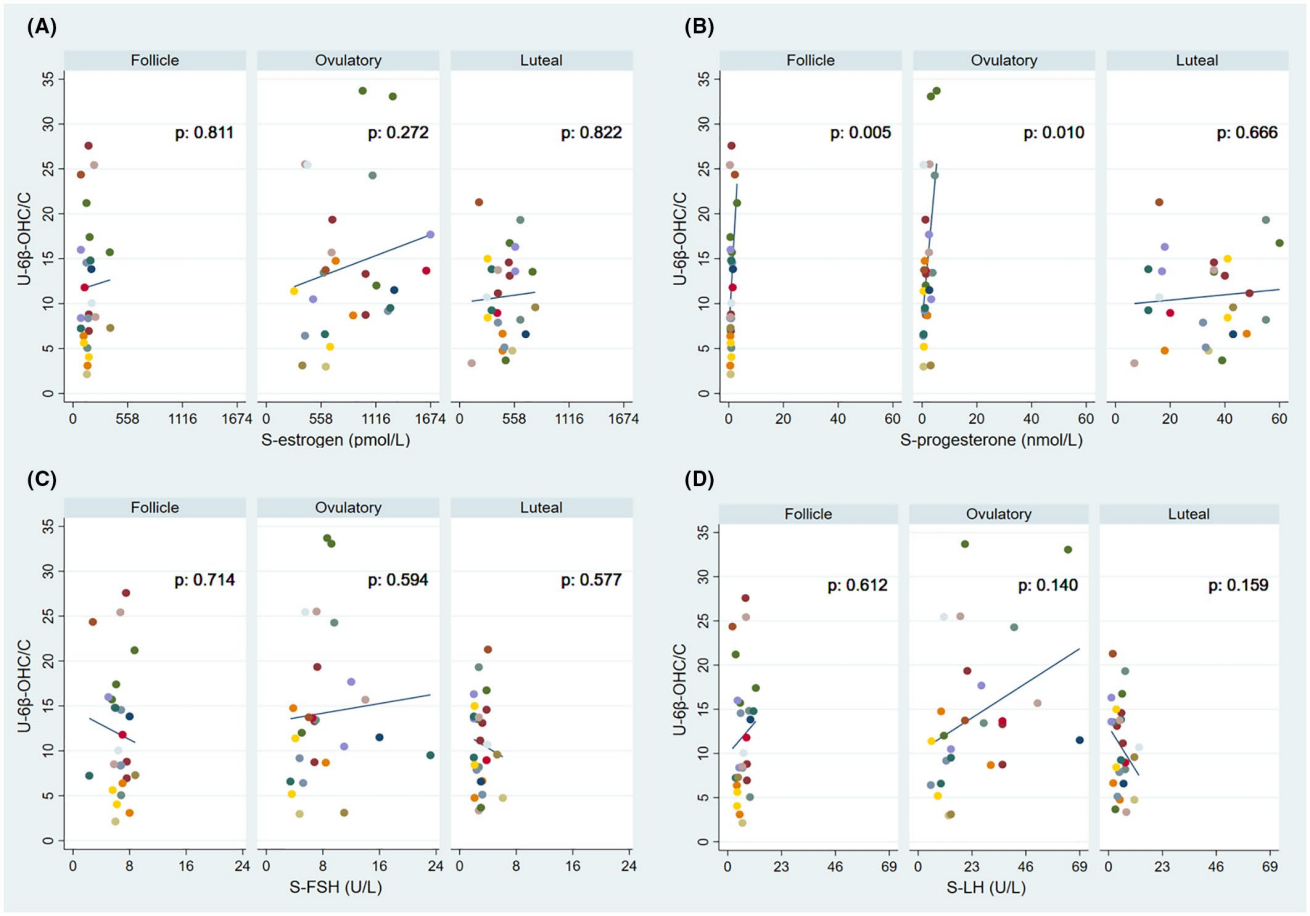
Linear regression analysis between 6β‐OH‐cortisol/cortisol (6β‐OHC/C) and (A) estrogen, (B) progesterone, (C) follicle stimulating hormone (FSH), and (D) luteinizing hormone (LH) during the follicular, ovulatory, and luteal phases of the menstrual cycle in 17 healthy women. Each subject is represented by a color, but as 11 subjects contributed with two complete cycles (six 6β‐OHC/C values) and four subjects with one cycle (three values), some colors are shown twice in each phase. A total of 78 values 6β‐OHC/C together represent 26 complete menstrual cycles (78/3 = 26)

Comparing the relationship between 6β‐OHC/C and sex hormones and gonadotropins during the menstrual phases (*n* = 26 for each phase), there was a significant association between the ratio and progesterone during the follicular (*p* = .005) and ovulatory (*p* = .01) phase (Figure 4).

**FIGURE 5:**
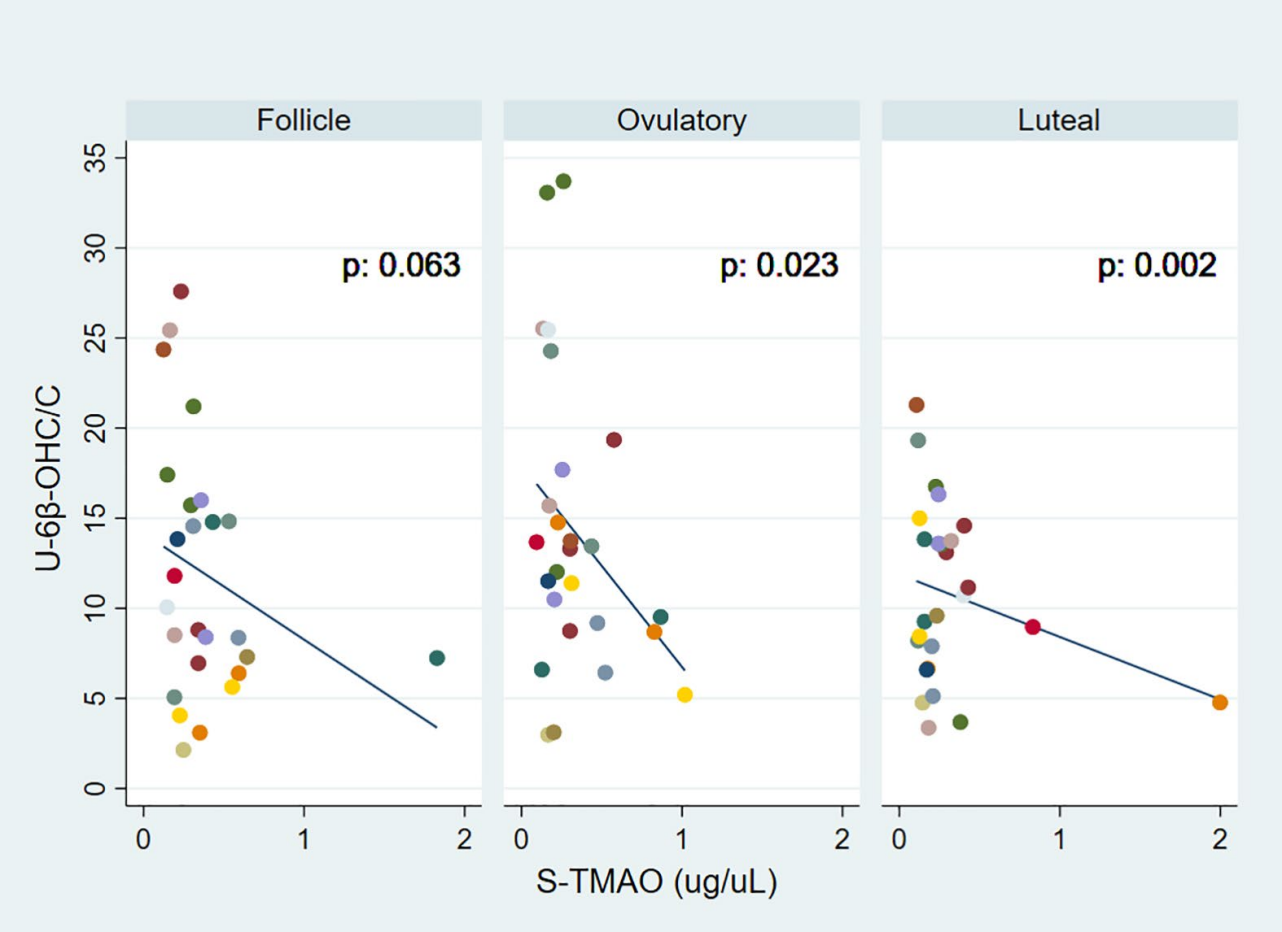
Linear regression analysis between 6β‐OH‐cortisol/cortisol (6β‐OHC/C) and the microbiota metabolite trimethylamine‐N‐Oxid (TMAO) during the follicular, ovulatory, and luteal phases of the menstrual cycle in 17 healthy women. Each subject is represented by a color, but as 11 subjects contributed with two complete cycles (six 6β‐OHC/C values) and four subjects with only one cycle (three values), some colors are shown twice in each phase. A total of 78 values 6β‐OHC/C together represent 26 complete menstrual cycles (78/3=26)

Similarly, an association was shown for 6β‐OHC/C and TMAO during the follicular (*p* = .063 ns), ovulatory (*p* = .023), and luteal (*p* = .002) phase (Figure 5). We have previously shown that there was no menstrual cycle variability in TMAO.^35^

**FIGURE 6:**
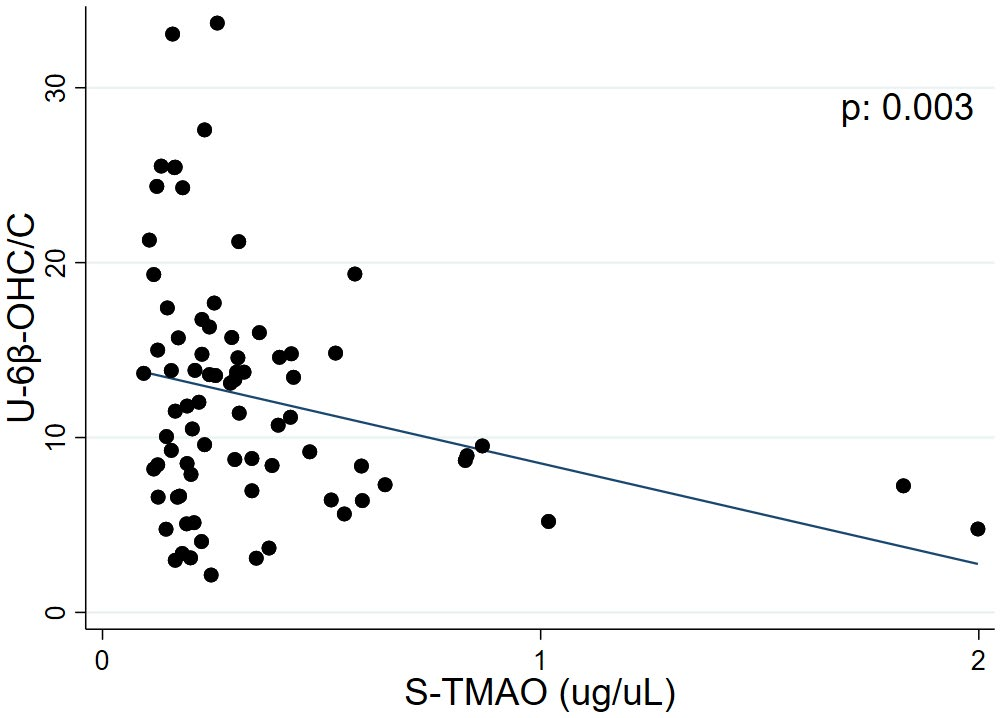
Linear regression analysis between all values, for all phases of 6β‐OH‐cortisol/cortisol (6β‐OHC/C) and the microbiota metabolite trimethylamine‐N‐Oxid (TMAO) during the menstrual cycle in 17 healthy women

Furthermore, there were no significant associations between 6β‐OHC/C and estrogen, progesterone or FSH when comparing all values, all phases (*n* = 78). For 6β‐OHC/C and LH, a border significant association (*p* = .055) was found (Figure S1). In addition, there was a significant association (*p* = .003) between 6β‐OHC/C and TMAO for all values, all phases (Figure 6).

### 3.4 MiRNA‐27b variability and association

**FIGURE 7:**
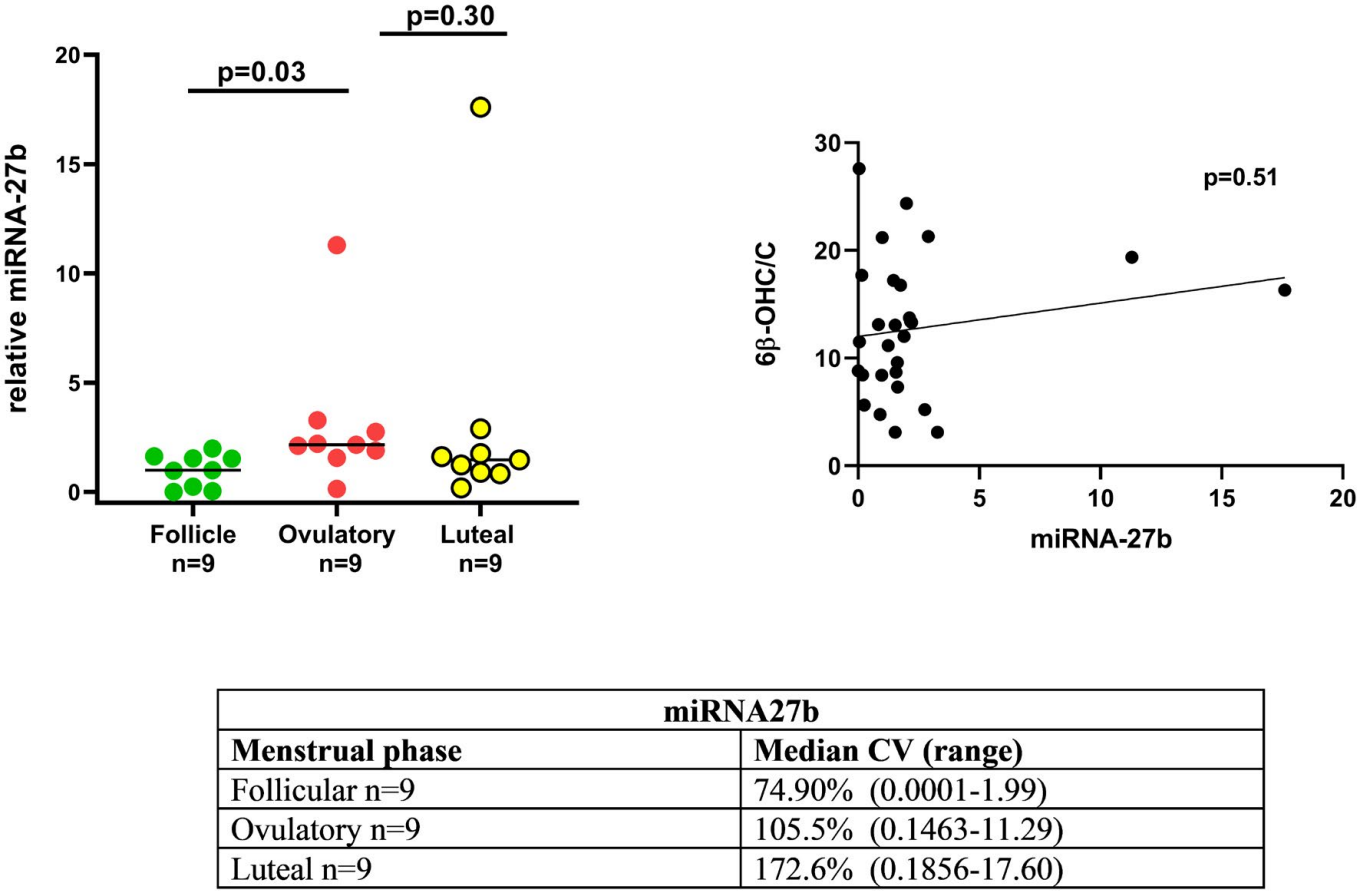
(A) Variations in miRNA‐27b during the three phases of the menstrual cycles from nine healthy volunteers (*n* = 27). (B) Correlation analysis between all values, for all phases of 6β‐OH‐cortisol/cortisol (6β‐OHC/C) and miRNA‐27b (*n* = 27)

The aim was to extract miRNA from one complete menstrual cycle including the three phases from all 17 subjects (*n* = 51). However, miRNA‐27b was only successfully extracted from 39 samples. For four subjects there were not enough serum samples available, and for three participants only two phases were present, and therefore excluded from the statistical analysis. Thus, nine complete menstrual cycles from nine subjects (*n* = 27) were included in the final analysis. There was a significant difference (*p* = .03) between the follicular and ovulatory phase in the relative miRNA‐27b expression (Figure 7). When evaluating the association between 6β‐OHC/C and miRNA‐b27 (*n* = 27) all phases, there was no correlation (*p* = .51). Finally, there was no correlation between miRNA27b and all values, all phases of estrogen (*p* = .96), progesterone (*p* = .96), FSH (*p* = .34), LH (*p* = .92), or TMAO (*p* = .67). The median CV for miRNA‐27b was 74.9% in the follicular phase, 105.5% in the ovulatory, and 172.6% in the luteal phase.

## 4 DISCUSSION

In this pilot study, no significant menstrual cycle variability in the constitutive urinary metabolic ratio of 6β‐OHC/C was found. The intra‐ and interindividual variability was in line with previous findings in women.^20^, ^22^ There was a significant difference in the relative expression of miRNA‐27b between the follicular and ovulatory phase. When evaluating one menstrual phase at the time, there was a significant association between 6β‐OHC/C and progesterone during the follicular and ovulatory phase. Similarly, an association between the ratio and TMAO during the ovulatory and luteal phase was shown. When analyzing all values, from all phases, there was a significant association between 6β‐OHC/C and microbiota TMAO and a border significant association with LH.

Compared to previous studies on 6β‐OHC/C during the menstrual cycle, the three different menstrual phases are more exactly defined in this study, based on hormone levels in the circulation. In addition, this is the first study evaluating how CYP3A activity in the basal state may be associated with the microbe metabolite TMAO and to miRNA27‐b during the menstrual cycle.

### 4.1 Differences in 6β‐OHC/C related to sex‐ and menstrual cycle phases

Although it has been suggested that estrogen regulate CYP3A expression in the liver,^40^, ^41^ investigations of sex difference in CYP3A activity measured as 6β‐OHC/C in urine have shown diverging results.^25^ While one study found the ratio to be higher in men,^19^ another study found the ratio to be higher in women.^42^ However, many studies of 6β‐OHC/C have been performed in males only.^43^, ^44^, ^45^

With regard to effects of the menstrual cycle on the 6β‐OHC/C in women, this has been considered in five studies.^19^, ^20^, ^21^, ^22^, ^23^ However, in one study, 4 of 10 subjects were using oral contraceptives or hormone replacement therapy,^22^ and in the remaining studies there were no significant menstrual cycle fluctuations. The strength of our study, compared to the previous studies, is the use of urine sampling for two complete menstrual cycles, in parallel with repeated hormone measurements in serum, in the same individuals (*n* = 17). In this manner, the determination of the different phases of the menstrual cycle was made more accurate than in previous studies.

In addition, disparities in methods analyzing cortisol and 6β‐OH‐cortisol makes it hard to compare values from different studies. Indeed, methods have varied from radioimmune‐ or enzyme immunoassay (RIA), to HPLC‐UV, LC‐MS/MS, and GC‐MS/MS^25^, ^38^, ^46^ Also, both single‐spot sample and 24‐hour urine collection has been used. Critics argue that the circadian rhythm of cortisol is not captured using spot sampling^47^ while others argue that a single morning sample adequately reflect the diurnal variability.^16^, ^22^ Finally, the 6β‐OHC/C varies with age and sex in the investigated population.^48^

### 4.2 6β‐OHC/C and progesterone and LH

Enzyme CYP3A is involved in the 6β‐hydroxylation of both cortisol and progesterone, so the association between 6β‐OHC/C and progesterone in our study, is not unexpected.^49^ Also, LH stimulates the secretion of progesterone and vice versa, and as such they are interrelated.^50^ The association between 6β‐OHC/C and progesterone was significant during the follicular and ovulatory phases. Progesterone is at its lowest during these phases, but levels rise sharply after the short (12–24 h) ovulation. The lack of an association between the ratio and progesterone during the luteal phase, could be due to changes in CYP3A enzyme activity or to changes in 6β‐OHC/C unrelated to CYP3A. In vitro data suggest that progesterone may affect the active, binding site of CYP3A though allosteric modification, and in this manner could change the substrate preference of CYP3A during the luteal phase.^51^

### 4.3 6β‐OHC/C and microbiota TMAO

In our study, the association between 6β‐OHC/C and TMAO reached significance for the ovulatory and luteal phases, when estrogen is higher compared to the follicular phase (Figure 1). In addition, high levels of TMAO were associated with decreased levels of 6β‐OHC/C, indicating decreased CYP3A activity.

The association between 6β‐OHC/C and TMAO, suggest that microbiota may affect drug metabolism.^29^, ^52^, ^53^ In fact, a reduction in the levels of CYP3A mRNA and protein in the liver was found in a study of germ‐free mice lacking intestinal microbiota, when compared to specific pathogen‐free mice.^54^ Interestingly, sex differences in the effects of the microbiome on mRNA expression of liver CYP3A have been found in mice.^55^

The formation of TMAO has been shown to be dependent on estrogen levels.^56^ Still, we have previously shown that the levels of TMAO did not vary significantly during the different menstrual phases.^35^ Moreover, there was no significant association between TMAO and estrogen, for all phases^35^ or during the three phases (unpublished data). Nonetheless, the phase‐specific association between TMAO and 6β‐OHC/C suggest that high levels of estrogen may be involved in the possible effect TMAO has on CYP3A activity.

Another possible mechanism, is that secondary bile acids produced by microbiota, act as signaling molecules by activating nuclear receptor Pregnane X receptor (PXR), a known regulator of CYP3A expression in the liver.^53^, ^57^ Interestingly, bile acids also activate Farnesoid X receptor (FXR), that regulate levels of TMAO.^57^, ^58^

Finally, the activity in the immune system, has been shown to vary during the menstrual cycle and inflammation has been shown to downregulate CYP expression in the liver.^59^, ^60^ From a theoretical point of view, activation of pro‐inflammatory cytokines like TNF‐α by TMAO, could modulate the activity of CYP3A, and thus explain the association.^27^, ^59^

### 4.4 miRNA‐27b

A significant difference in miRNA‐26b between follicular and ovulatory menstrual phase was noted, and this finding should be investigated in larger studies.

In a recent study of 105 male patients with alprazolam treatment due to anxiety disorder, a significant association was found between 6β‐OHC/C and miRNA‐27b.^61^ However, there was no association between 6β‐OHC/C and miRNA‐27b in this study. This may be due to differences between the sexes or to the different roles miRNAs may have in biological functions and diseases.^62^, ^63^ It is also possible that the sample size was too small to detect differences in this study cohort, that is, a type II error. The reproducibility of miRNA results may also be confounded by the choice of endogenous control.^31^ Herein miR‐26b was chosen as a control miRNA and was proven to be highly abundant in the serum with stable Ct values not dependent on the menstrual phases (data not shown).

### 4.5 Implications of studies on menstrual cycle and CYP3A activity

Although a sample size of 17 individuals may appear small, most studies on menstrual cycle variability of CYP3A markers have small samples sizes, from four to an average of 20 individuals.^7^, ^11^, ^13^ One reason for this, could be that studies involving administration of drugs causing inhibition or induction, in combination with phenotyping with midazolam iv and/or orally, may be difficult to perform in vulnerable populations like women of child‐bearing age, in elderly and in children.^21^, ^22^ Unfortunately, investigations have shown that women are still very much underrepresented in DDI studies, and it has been suggested that the use of endogenous markers of CYP3A could increase the number of women in DDI studies.^32^, ^64^

### 4.6 Limitations of this study

The limitations of our pilot study are several. As previously mentioned, the method of urine sampling may have had an impact on the results. In addition, due to the small sample size, we did not use genotyping to evaluate potential effects of genetic polymorphisms on our results. Finally, although there are data supporting that serum miRNA‐27b may relate to hepatic miRNA‐27b, this finding has yet to be confirmed in the same subjects.^31^ However, obtaining liver tissue samples from healthy volunteers will most likely turn out to be a difficult task.

## 5 CONCLUSION AND FUTURE DIRECTIONS

In conclusion, we did not find evidence supporting the hypothesis of a variability in CYP3A activity measured by 6β‐OHC/C, during the three menstrual cycle phases. Whether the association between 6β‐OHC/C and progesterone, will have any implications for the use of 6β‐OHC/C as a marker of CYP3A activity in fertile women—remains to be explored. Interestingly, the finding of an association between 6β‐OHC/C in urine and levels of TMAO in serum suggest that gut microbiota may affect CYP3A activity.

In the future, more thorough studies on how the gut microbiota may affect CYP3A activity in women and men are needed. It has recently been suggested that 6β‐OHC/C represents CYP3A activity in the liver only.^44^ Thus, investigations of markers reflecting the CYP3A activity in the gut would also be of interest, and how this activity may be affected by gut microbiota as well as by sex hormones.

## DISCLOSURE

The authors declare that they have no conflict of interest.

## AUTHOR CONTRIBUTIONS

Linda Björkhem‐Bergman, Lena Ekström, and Helena Bergström were responsible for study design, funding applications, application to Human Ethics Committee of Karolinska Institutet, Biobank of Sweden and Swedish Data Protection Authority. Recruiting and screening of subjects was performed by Linda Björkhem‐Bergman as study physician and Lena Ekström. Ulf Diczfalusy and Anna Lindahl were responsible for method and analysis of 6β‐OHC/C. Helena Bergström compiled data from laboratory reports and CRFs. Helena Bergström performed statistical analysis together with biostatistician Anna Warnqvist and Linda Björkhem‐Bergman. All authors contributed to the manuscript. All authors read and approved the final manuscript.

## ETHICS APPROVAL STATEMENT

The study was approved by the Human Ethics Committee of Karolinska Institutet (Dnr 2018/481‐31/2 and Dnr 2020‐00598). The study was performed in accordance with the Declaration of Helsinki. Written informed consent was obtained from all patients prior to inclusion in the study.

## Supporting information

Figure S1

Figure S2

Table S1

## Data Availability

As sample size is small, there is a risk for the individual privacy being compromised. De‐identified data are therefore available from the corresponding author upon reasonable requests.
