# Supplementary figures and images for: Studies on CYP3A activity during the menstrual cycle as measured by urinary 6β‐hydroxycortisol/cortisol
Source: Pharmacol Res Perspect. 2021 Oct 19;9(6):e00884. doi: 10.1002/prp2.884 (PMC8525181; doi:10.1002/prp2.884)

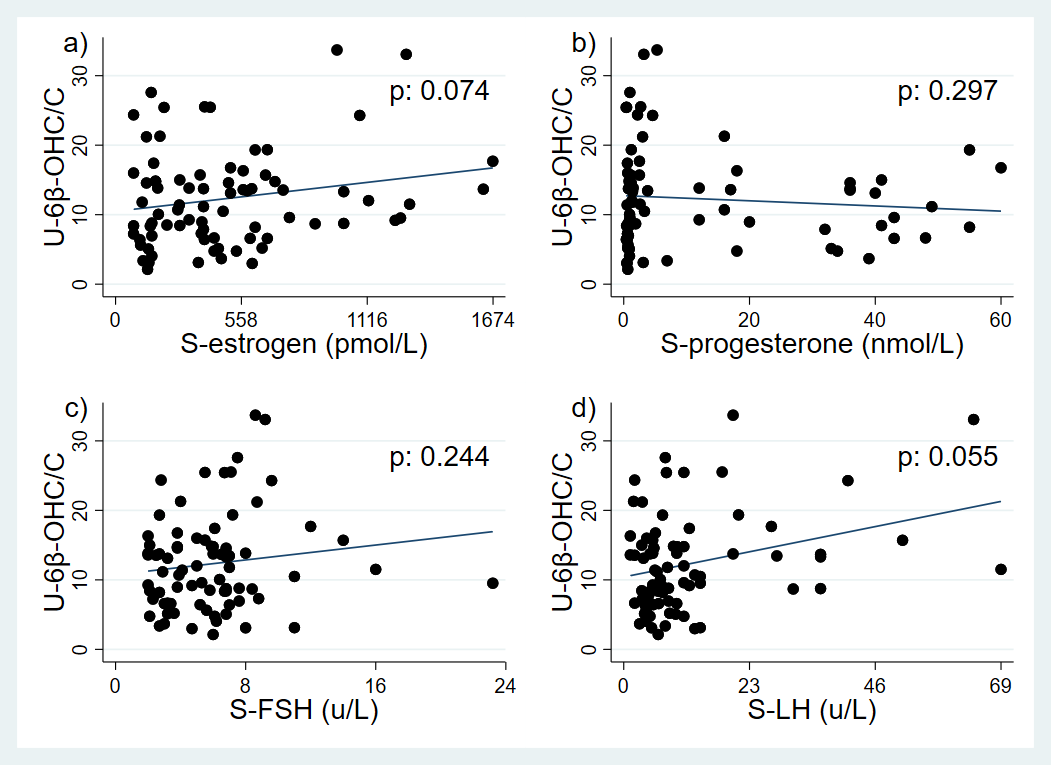

Supplement: Supplementary file 1 — Figure S1 [file PRP2-9-e00884-s003.tif]

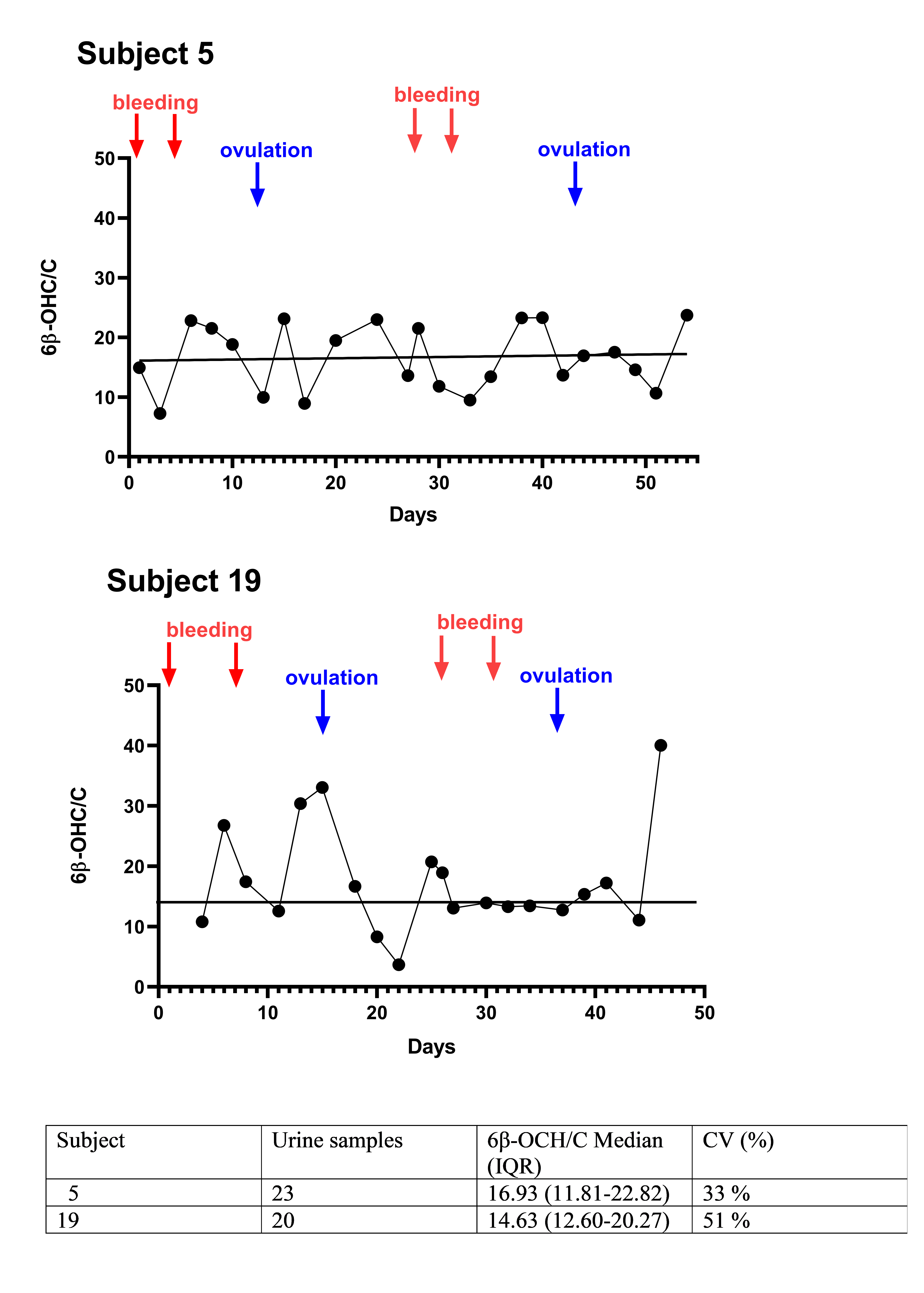

Supplement: Supplementary file 2 — Figure S2 [file PRP2-9-e00884-s001.tif]
