# Supplementary material for: Studies on CYP3A activity during the menstrual cycle as measured by urinary 6β‐hydroxycortisol/cortisol
Source: Pharmacol Res Perspect. 2021 Oct 19;9(6):e00884. doi: 10.1002/prp2.884 (PMC8525181; doi:10.1002/prp2.884)
Supplement: Supplementary file 3 — Table S1 [file PRP2-9-e00884-s002.docx]

| Subject | urine samples (n) | 6βOHC/C Median (IQR) | CV (%) |
| --- | --- | --- | --- |
| 1 | 6 | 10.96 (8.30-13.63) | 29 % |
| 2 | 6 | 16.24 (13.16-23.32) | 42 % |
| 3 | 6 | 6.52 (4.35-10.21) | 55 % |
| 4 | 6 | 14.14 (7.42-20.56) | 45 % |
| 5 | 6 | 12.74 (10.25-18.90) | 37 % |
| 6 | 6 | 14.80 (9.97–16.66) | 27 % |
| 7 | 3 | 2.98 (2.14-4.76) | 41 % |
| 8 | 3 | 21.29 (13.74-24.36) | 28 % |
| 9 | 6 | 8.13 (6.10-10.53) | 38 % |
| 10 | 6 | 9.40 (7.08-14.07) | 33 % |
| 12 | 3 | 7.30 (3.12-9.59) | 49 % |
| 13 | 6 | 14.72 (7.23-25-45) | 58 % |
| 14 | 6 | 7.04 (4.91-12.30) | 51 % |
| 15 | 6 | 10.83 (9.36-16.85) | 49 % |
| 16 | 6 | 11.58 (7.25-12.23) | 27 % |
| 17 | 3 | 19.95 (11.16-27.59) | 42 % |
| 19 | 6 | 15.56 (10.99-21.33) | 58 % |
|  | N=90 |  | 42 %* |

*Mean values
Coefficient of variation (CV). Interquartile range (IQR), 6β-hydroxycortisol to cortisol (6βOHC/C)
